# Timely initiation of breastfeeding among women who gave birth by cesarean section in central Ethiopia, 2022: A cross-sectional study

**DOI:** 10.1371/journal.pone.0291983

**Published:** 2023-09-27

**Authors:** Arega Abebe Lonsako, Haymanot Mezmur, Arsema Gebreyesus, Gadissa Tolosa, Sagni Girma

**Affiliations:** 1 School of Nursing, College of Medicine and Health Science, Arba Minch University, Arba Minch, Ethiopia; 2 School of Nursing and Midwifery, College of Health and Medical Science, Haramaya University, Harar, Ethiopia; 3 Department of Obstetrics and Gynecology, Leiden University Medical Centre, Leiden, the Netherlands; Arba Minch University, ETHIOPIA

## Abstract

**Background:**

Timely initiation of breastfeeding reduces the risk of neonatal mortality. However, there was paucity of literature on the timely initiation of breastfeeding among women who gave birth by cesarean section (CS) in Ethiopia. Thus, the aim of this study was to assess the magnitude of timely initiation of breastfeeding and factors associated with it among women who gave birth by CS in central Ethiopia.

**Methods:**

A facility-based cross-sectional study was conducted among 403 women who gave birth by CS. Data were collected by using an interviewer-administered questionnaire and observation checklist, entered into EpiData 4.6, and exported to statistical package for the social sciences (SPSS) version 26.Descriptive and multivariate logistic regression analyses were performed and statistical significance is declared at *p*<0.05.

**Results:**

The magnitude of timely initiation of breastfeeding was 47.4% [95% CI: (42.5, 52.6)]. Attending four or more antenatal care visits [(AOR): 2.27, 95%CI: (1.28, 4.02)], counseling during antenatal care [AOR: 4.78, 95% CI: (2.66, 8.60)], early skin to skin contact with newborn [AOR: 2.83, 95% CI: (1.60, 5.02)], post-delivery counseling [AOR: 2.93, 95% CI: (1.56, 5.50)], and getting assistance from health professionals [AOR: 3.07, 95% CI: (1.64, 5.75)] were factors associated with timely initiation of breastfeeding.

**Conclusions:**

The magnitude of timely initiation of breastfeeding in the study area was low. Strengthening counseling by health care practitioners during ANC and post-natal period should be prioritized to support women in initiating early skin-to-skin contact within one hour of birth is mandatory.

## Introduction

Breastfeeding is one of the most practical and inexpensive approaches to reducing infant mortality and morbidity [1]. The World Health Organization (WHO) and the United Nations International Children’s Emergency Fund (UNICEF) recommended the timely initiation of breastfeeding [2]. Timely initiation of breastfeeding (TIBF) is starting breastfeeding for newborns within one hour of delivery [3], including colostrum feeding which is richer in nutrients and antibodies [4].

TIBF has a role in the survival and health of both mother and newborn. For the newborn, it creates a special bond with mother, improves cognitive development, decreases the risk of infection and sudden infant death syndrome while for the mother, the baby’s suckling stimulates the production of hormones, which motivate the uterus to contract, and reduce the risk of postpartum bleeding, earlier return to pre-pregnancy weight, prevents breast engorgement, and reduces the risk of breast cancer later in her life [3,5]. It is one of the approaches to reduce neonatal mortality globally [6] which contributes to nearly half of under-five mortality [7]. It has been shown to reduce neonatal mortality by 22% [8]. Newborns who started breastfeeding after one hour of birth were 33% more at risk for mortality [9].

Cesarean sections (CS) delivery significantly increased throughout the world, accounting for 21.1% of deliveries [10]. In sub-Saharan Africa (SSA), it varies from 2% to 51% of deliveries [11], and in Ethiopia for 29.55% of deliveries [12], which was higher than the ideal rate of cesarean section recommended by WHO (10–15%) [13]. CS was considered to be a barrier to the TIBF, although some studies revealed that it is possible to initiate breastfeeding within one hour of cesarean delivery under spinal anesthesia [14].

Despite its importance to reduce risk of neonatal mortality, only 51.9% of mothers in the south Gondar zone [15], 57% in Dessie city [16], and 51.2% in Addis Ababa [17], mothers who gave birth by CS, practiced TIBF in Ethiopia, which was lower than the national target (92%) [18].

Previous studies done in Ethiopia on TIBF focused mainly among women who gave birth by spontaneous vaginal delivery and limited studies were conducted so far among cesarean section deliveries. In addition, there is no documented study in health facilities of central Ethiopia in this subject matter. Therefore, this study aims at assessing the timely initiation of breastfeeding and its associated factors among women who gave birth by cesarean section at hospitals of Kembata Tembaro and Hadiya zones in central Ethiopia.

## Methods and materials

### Study design and area

We conducted a facility-based cross-sectional study in hospitals of Kembata Tembaro and Hadiya zones in central Ethiopia from May 01 to 30, 2022. Kembata Tembaro and Hadiya zones are two of the five zones in central Ethiopia region, having a total population of 1,080,837and 1,727,920 respectively. Which are 272 km and 230 km away from the capital city of Ethiopia, Addis Ababa respectively. The estimated annual delivery was 30,286 in Kembata Tembaro zone and 23,153 in Hadiya zone. There are four hospitals that provides cesarean delivery services, 36 health centers and 138 health posts in Kembata Tembaro zone while in Hadiya zone there are four hospitals that provide cesarean delivery, 61 health centers, and 311 health posts [19].

### Sample size determination and sampling techniques

We calculated sample size by using a single population proportion formula with the assumptions; the margin of error (d) = 5%, confidence level = 95% (Zα/_2_ = ±1.96), and the proportion of TIBF (p) among mothers who gave birth by cesarean section was 57% [16]. So, the final calculated sample size with a 10% non-response rate was 415.

All hospitals that provide cesarean delivery services in Kembata Tembaro and Hadiya zones were included. The sample was allocated proportionally based on past CS deliveries in the identified hospitals. Wachemo University Nigist Elleni Mohamed Memorial Comprehensive Hospital (192), Shone Primary Hospital (25), Hommacho Primary Hospital (20), Gimbichu Primary Hospital (18), Shinshicho Primary Hospital (35), Mudula Primary Hospital (28), Doyogana Primary Hospital (22), and Dr. Bogalech Gebre Memorial General Hospital (75). Finally, a consecutive sampling was used to select women who gave birth by cesarean section to get the desired sample size.

The source population were all mothers who gave birth by CS in hospitals of Kembata Tembaro and Hadiya zones, while all mothers who gave birth by cesarean section in hospitals of Kembata Tembaro and Hadiya zones during the data collection period were considered as the study population.

We included women who gave birth by cesarean delivery under spinal anesthesia in hospitals of Kembata Tembaro and Hadiya zones, whereas women whose newborns were admitted to the neonatal intensive care unit (NICU) immediately after delivery were excluded from the study.

### Data collection tool and procedures

Data collection tools were adapted from reviewing related literatures [20], and WHO indicators to measure timely initiation of breastfeeding. It consists of socio-demographic characteristics, information about timely initiation of breastfeeding and colostrum feeding, obstetric and health service related characteristics, maternal obstetric and medical complications related questions, timely initiation of breastfeeding questions and newborn factors related questions. The questionnaire was prepared in English and translated to Amharic and local languages (Kembatisa and Hadiyisa) and back to English by a language expert to check its consistency.

Data were collected by eight bachelor nurses and supervised by four supervisors (two master degree holders in maternity nursing and two bachelors in public health). Brief orientation was given to respondents on the purpose of the study and thus volunteers were interviewed face-to-face and their charts were reviewed. After screening the area for privacy, the eligible women were interviewed at their bedsides within 72 hours of cesarean delivery before requesting discharge from the hospital, and detailed information was collected.

The study’s explanatory variables include Age, Level of education, Employment, Place of residence, Number of antenatal care visits, Type of pregnancy, Counseling about timely initiation of breastfeeding during ANC follow-up, Post-cesarean section counseling, Obstetric complications, Assistance from health professionals for timely initiation of breastfeeding, Early skin-to-skin contact with a newborn baby, Informed about timely initiation of breastfeeding, Breastfeeding experiences, Prelacteal feeding, Birth weight, APGAR score and Sex of the newborn.

### Operational definitions

**Timely initiation of breastfeeding**: is the initiation of breastfeeding for newborn within one hour of birth. It was calculated based on responses to the question “How long after delivery did you start breastfeeding for your newborn?” The responses were recorded in hours and a binary variable of timely initiation of breastfeeding was generated (Yes = 1, if the initiation of breastfeeding was within an hour of birth, No = 0, if the initiation of breastfeeding was after one hour of birth) [15,16].

**Health professionals’ assistance for timely initiation of breastfeeding:** is assisting the women and newborn baby for proper positioning and attachment to initiate breastfeeding within one hour of delivery.

**Prelacteal feeding:** is feeding any liquid or solid food other than mother’s breast milk before initiating breastfeeding to the newborn baby [21].

### Data quality control

To maintain the data quality, the two-days training was given to data collectors and supervisors about the objective of the study, ethical issues, and data collection methods. Tools were translated to local languages and a pretest was conducted on 5% of the total sample size at Halaba Kulito General Hospital. Based on the results of the pretest, adjustments were made as needed, and the amended tool was used for actual data collection. Supervisors closely followed the data collectors and data collectors were instructed to check the completeness of the questionnaire immediately after its completion for each participant.

### Data analysis

The data were entered using EpiData 4.6 and analyzed using SPSS version 26. Descriptive statistics were used to describe the participants, and the results were presented in the form of text, tables, and figures. Binary logistic regression model was used to identify the association between each independent variable and the outcome variable. In bivariate analysis, independent variables with p-value less than 0.25 were selected for multivariable analysis. Multi-collinearity was checked by using the variance inflation factor and standard error. The model fitness was checked by the Hosmer Lemeshow goodness of fit test. Adjusted Odds Ratio (AOR) with a 95% confidence interval (CI) was reported, and p-value <0.05 were considered as significantly associated with the timely initiation of breastfeeding.

### Ethical considerations

Ethical clearance was obtained from Haramaya University College of Health and Medical Sciences Institutional Health Research Ethics Review Committee (Reference number: IHRERC/064/2022). A letter of support was written to the study hospitals. Before starting the data collection, the medical directorates of each hospital provided their written and signed permission. Each participant was informed of the study’s purpose, potential benefits, risks, and their right to decide whether to participate in the study before enrolling in it. Then informed voluntary written and signed consent was obtained from each participant. Assents were sought from participants under the age of 18, and their parents or legal guardians provided written informed consent. The participants’ names were neither sought nor recorded in order to maintain confidentiality.

## Results

### Socio-demographic characteristics of participants

A total of 403 women who gave birth by cesarean section participated in this study, with a response rate of 97.1%. The median age of the women was 29 years with an interquartile range (IQR) of 27 to 32 years. Of all, 168(41.7%) women were in the age group of 25–29 years and 232(57.6%) were urban dwellers. All women, 403(100%) were married, and 222(55.1%) were protestant (Table 1).

**Table 1:** Socio-demographic characteristics of women who gave birth by CS at hospitals of Kembata Tembaro and Hadiya zones in southern Ethiopia, 2022 (n = 403).

Variable	Category	Frequency (n)	Percent (%)
Age in years	15–19	4	1.0
	20–24	52	12.9
	25–29	168	41.7
	30–34	157	39.0
	≥ 35	22	5.4
Educational status	No formal education	18	4.5
	Primary school	147	36.5
	Secondary school	154	38.2
	College and above	84	20.8
Occupation	Government employee	86	21.3
	NGO employee	3	0.7
	Merchant	106	26.3
	Housewife	207	51.4
Religion	Orthodox	151	37.5
	Protestant	222	55.1
	Muslim	30	7.4
Residence	Urban	232	57.6
	Rural	171	42.4
Husband’s educational status	No formal education	10	2.5
	Primary school	92	22.8
	Secondary school	87	21.6
	College and above	214	53.1

### Obstetric and health service-related characteristics

In this study, 403 (100%) of the women reported that they had a history of ANC follow-up in the current (index) pregnancy, even though, 211(52.4%) of them had less than four ANC visits and 223(55.3%) of women reported they had no counseling about timely initiation of breastfeeding during their ANC visits. Regarding the place of ANC follow up 223(55.3%) of women visited in hospitals, while 180(44.7%) visited in health centers, and only 140 (34.7%) received assistance from health professionals for timely initiation of breastfeeding after delivery. In terms of the CS type, 353(87.6%) of women had an emergency CS, and only 136 women (33.7%) received post-CS counseling about timely initiation of breastfeeding, and 227(56.3%) of women started early skin-to-skin contact with their newborn baby (Table 2).

**Table 2:** Obstetric and health service-related characteristics of women who gave birth by CS at hospitals of Kembata Tembaro and Hadiya zones, southern Ethiopia, 2022(n = 403).

Variables	Category	Frequency (n)	Percent(%)
Parity	Primipara	149	37.0
	Multipara	254	63.0
History of stillbirth	Yes	5	1.2
	No	398	98.8
History of abortion	Yes	17	4.2
	No	386	95.8
Number of ANC visits	< 4	211	52.4
	≥ 4	192	47.6
Counseling about TIBF during ANC visits	Yes	180	44.7
	No	223	55.3
Duration of labor in hours	≤ 12	259	64.3
	> 12	144	35.7
Type of cesarean section	Elective	50	12.4
	Emergency	353	87.6
Time of CS delivery	Day	274	68.0
	Night	129	32.0
Post-CS counseling about TIBF	Yes	136	33.7
	No	267	66.3
Early skin-to-skin contact with newborn	Yes	227	56.3
	No	176	43.7
Health professionals assistance to TIBF	Yes	140	34.7
	No	263	65.3
Obstetric complication	Yes	36	8.9
	No	367	91.1

TIBF: Timely Initiation of Breastfeeding, ANC: Antenatal Care, CS: Cesarean Section.

### Information about timely initiation of breastfeeding and colostrum feeding

In this study, 399(99.0%) reported they had heard about the TIBF, and 245(61.4%) of the women mentioned health professionals as a source of information. Around 368(91.3%) of women were aware of the importance of colostrum feeding for the newborns but only 276(68.5%) of the women feed it to their newborns (Table 3).

**Table 3:** Information about timely initiation of breastfeeding and colostrum feeding among women who gave birth by cesarean section at hospitals of Kembata Tembaro and Hadiya zones in southern Ethiopia, 2022(n = 403).

Variable	Category	Frequency(n)	Percent(%)
Informed about timely initiation of breastfeeding	Yes	399	99.0
	No	4	1.0
Source of information(n = 399)	Health professionals	245	61.4
	Neighbors/relatives	141	35.3
	Media	13	3.3
Colostrum is important for newborn	Yes	368	91.3
	No	35	8.7
Colostrum is given to the newborn	Yes	276	68.5
	No	127	31.5
Breastfeeding experience	Yes	254	63.0
	No	149	37.0
Pre lacteal feeding	Yes	32	7.9
	No	371	92.1

### Newborns characteristics

Over half, 238 (59.1%) of newborn babies were male, and nearly all, 398 (98.8%) of were delivered at term, while 5(1.2%) were late preterm (34–37 weeks). Majority, 325(80.6%) had Apgar score ≥7, while 78(19.4%) had Apgar score < 7 at the first minute of birth. In terms of birth weight 8(2%), 366 (90.8%), and 29(7.2%) of the newborns were delivered with a low birth weight (< 2.5 kg), normal birth weight (≥2.5 kg to <4 kg), and macrosomia (≥4 kg) respectively.

### The magnitude of timely initiation of breastfeeding

Themagnitude of timely initiation of breastfeeding among women who gave birth by cesarean section under spinal anesthesia in hospitals of Kembata Temabaro and Hadiyazones was 47.4% with 95% CI:(42.5, 52.6%).

### Factors associated with timely initiation of breastfeeding

After controlling for possible confounding factors having four or more ANC visits, counseling about TIBF during ANC follow-up, post-delivery counseling about TIBF, early skin-to-skin contact with newborn, and assistance from health professional’s during the initiation of breastfeeding were significantly associated with TIBF at 95% confidence level with *p*< 0.05.

Women who attended four or more antenatal care visits were 2.27 times more likely to initiate breast feeding within 1h of birth than who had less than four visits [(AOR): 2.27, 95%CI: (1.28, 4.02)]. Women who obtained counseling during antenatal care on TIBF were 4.78 times more likely to initiate breast feeding within 1h of birth than who did not get counseling [AOR: 4.78, 95% CI: (2.66, 8.60)]. Women who practice early skin to skin contact with newborn were 2.83 times more likely to initiate breast feeding than women who did not practice it [AOR: 2.83, 95% CI: (1.60, 5.02)], in addition women who received professional counseling during post-delivery were 2.93 times more likely to practice TIBF than who did not got [AOR: 2.93, 95% CI: (1.56, 5.50)]. Compared to women who did not got professional assistant from health care provider at the time of post-natal time those who got assistance from health professionals were 3.07 times more likely to initiate breast feeding within 1h of birth [AOR: 3.07, 95% CI: (1.64, 5.75)] (Table 4).

**Table 4:** Factors associated with timely initiation of breast feeding among women who gave birth by CS at hospitals of Kembata Tembaro and Hadiya zones in southern Ethiopia, 2022 (n = 403).

Variables	TIBF		COR(95%CI)	AOR(95%CI)	*p*-value
	Yes (%)	No (%)			
Place of residence					
Urban	131(32.5)	101(25.1)	2.39(1.59, 3.60)	1.37(0.79, 2.39)	0.260
Rural	60(14.9)	111(27.5)	1.00	1.00	
Number of ANC visits					
≥ 4	137(34.0)	55(13.6)	7.24(4.66, 11.24)	**2.27(1.28, 4.02)** *	**0.005**
< 4	54(13.4)	157(39.0)	1.00	1.00	
Breastfeeding experience					
Yes	141(35.0)	113(28.0)	2.47(1.62, 3.76)	1.22(0.61, 2.44)	0.570
No	50(12.4)	99(24.6)	1.00	1.00	
Counseling about TIBF during ANC					
Yes	141(35.0)	39(9.7)	12.50(7.78, 20.09)	**4.78(2.66, 8.60)** *	**0.001**
No	50(12.4)	173(42.9)	1.00	1.00	
Duration of labor in hours					
≤ 12	143(35.5)	116(28.8)	2.46(1.61, 3.76)	1.22(0.61, 2.45)	0.561
> 12	48(11.9)	96(23.8)	1.00	1.00	
Post-CS counseling about TIBF					
Yes	103(25.6)	33(8.2)	6.34(3.97, 10.13)	**2.93 (1.56, 5.50)** *	**0.002**
No	88(21.8)	179(44.4)	1.00	1.00	
Type of cesarean section					
Elective	37(9.2)	13(3.2)	3.67(1.88, 7.15)	0.65(0.22, 1.93)	0.444
Emergency	154(38.2)	199(49.4)	1.00	1.00	
Early skin-to-skin contact					
Yes	151(37.5)	76(18.9)	6.75(4.31, 10.56)	**2.83(1.60, 5.02)** *	**0.001**
No	40(9.9)	136(33.7)	1.00	1.00	
Time of CS delivery					
Day	146(36.2)	128(31.8)	2.12(1.38, 3.28)	1.04(0.57, 1.90)	0.877
Night	45(11.2)	84(20.8)	1.00	1.00	
Assistance from Health Professionals for TIBF					
Yes	113(28.0)	27(6.7)	9.92(6.04, 16.30)	**3.07(1.64, 5.75)** *	**0.001**
No	78(19.4)	185(45.9)	1.00	1.00	

**Key**-TIBF: Timely Initiation of Breastfeeding, COR: Crude Odd Ratio, AOR: Adjusted Odd Ratio,1.00: Reference group,

* = shows significance.

## Discussion

This study assessed the magnitude of timely initiation of breastfeeding and its associated factors among women who gave birth by cesarean section. The magnitude of timely initiation of breastfeeding in this study setting was 47.4%. Four or more ANC visits, counseling about TIBF during ANC follow-up, post-delivery counseling about TIBF, early skin-to-skin contact with newborn baby, and assistance from health professionals for TIBF were significantly associated with timely initiation of breastfeeding.

The magnitude of timely initiation of breastfeeding in this study was consistent with the finding of studies conducted in the south Gondar zone, Ethiopia (51.9%) [15], and Addis Ababa, Ethiopia (51.2%) [17]. However, this finding was lower than the result of studies done in Dessie city, Ethiopia (57%) [16], Malaysia (73.7%) [22], and WHO global survey (57.6%) [23]. This discrepancy might be due to difference in sampling procedure because we used consecutive sampling technique whereas they used systematic sampling technique. And this finding was higher than the results of the studies conducted in Kenya (25%) [24], and Egypt (28.1%) [25]. This variation might be due to differences in the inclusion criteria (they included women who gave birth under general anesthesia, which delays the initiation of breastfeeding), study setting, and the time that the study was done.

In this study, women who had four or more ANC visits were 2.27 times more likely to initiate breastfeeding within one hour of delivery than those who have less than four ANC visits. This finding was supported by the study conducted in the south Gondar zone, Ethiopia [15], Indonesia [26], Ghana [27], and Bangladesh [28]. The possible reasons could be women who had frequent antenatal care visits during their pregnancy could get more chance of getting awareness about timely initiation of breastfeeding and its importance on newborn baby survival, which would help them to practice timely initiation of breastfeeding upon delivery [29].

The odds of timely initiation of breastfeeding were 4.78 times higher among women who received counseling about TIBF during their ANC follow-up than their counterparts. This finding was in line with a study done in Dessie city, Ethiopia [16], Brazil [30], and India [31]. This might be because counseling about timely initiation of breastfeeding during ANC follow-up provides information about the effect of timely initiation of breastfeeding on mother and newborn health, which motivates the mothers to plan timely initiation of breastfeeding for their newborn baby [32].

Furthermore, the odds of timely initiation of breastfeeding were 2.83 times higher among women who practiced early skin-to-skin contact with their newborns when compared with women who didn’t apply early skin-to-skin contact with their newborns. This finding was similar to a study conducted in Malaysia [22], and Nepal [33]. This might be due to the reason that most newborns were ready to find the nipple and latch on to the breast immediately after birth, if early skin-to-skin contact between mother and newborn is implemented, naturally the newborns have a tendency toward the breast [34].

Women who received post-cesarean section counseling about TIBF were 2.93 times more likely to initiate breastfeeding within one hour of delivery than those who didn’t get post-CS counseling. This finding was consistent with a study conducted in Dessie city, Ethiopia [16], and India [31]. This might be because of immediate, attentive advice and information from obstetric care providers about the importance of timely initiation of breastfeeding and its role on both maternal and newborn health, mothers are more likely to initiate breastfeeding within one hour of delivery.

In addition, the odds of timely initiation of breastfeeding were 3.07 times higher among women who got assistance from health professionals for timely initiation of breastfeeding when compared with women who didn’t get assistance from health professionals. This finding was supported by the study done in the south Gondar zone, Ethiopia [15], Addis Ababa, Ethiopia [17], Bangladesh [35], and Uganda [36]. This might be due to assistance from obstetric care providers for proper attachment and positioning to initiate breastfeeding within one hour of delivery, mothers get emotional well-being and inspiration, which help them to initiate breastfeeding within one hour of delivery [37].

Due to the lack of significant research on this subject and the fact that participants were not selected at random, it is impossible to draw general conclusions from this institution-based study; comparability is also challenging, which were the limitations of our study.

## Conclusions

The magnitude of timely initiation of breastfeeding among women who gave birth by cesarean section delivery in the study area was low compared to the national target for timely initiation of breastfeeding (92%). Attending four or more antenatal care visits, counseling about timely initiation of breastfeeding during ANC follow-up, assistance from health professionals for timely initiation of breastfeeding, early skin-to-skin contact with newborn baby, and post-delivery counseling were factors significantly associated with timely initiation of breastfeeding. Focus should be given to assist women to complete the antenatal care follow up, start early skin to skin contact with their newborn baby and initiate breastfeeding within one hour of delivery through strengthening the counseling about timely initiation of breastfeeding during ANC follow up and immediate postnatal period by health care providers.

## Supporting information

S1 Dataset The data set used to produce the study’s findings. (XLSX)

S1 File Data collection questionnaire in English language. (DOCX)

## Abbreviations

TIBF: Timely Initiation of Breastfeeding
ANC: Antenatal Care
AOR: Adjusted Odds Ratio
CI: Confidence Interval
SPSS: Statistical Package for Social Science

## References

[1] Victora CG, Bahl R, Barros AJ, França GV, Horton S, Krasevec J, et al. Breastfeeding in the 21st century: epidemiology, mechanisms, and lifelong effect. Lancet (London, England). 2016;387(10017):475–90. doi: 10.1016/S0140-6736(15)01024-7 26869575

[2] WHO. WHO Guidelines Approved by the Guidelines Review Committee. Guideline: Protecting, Promoting and Supporting Breastfeeding in Facilities Providing Maternity and Newborn Services. Geneva: World Health Organization; 2017. 29565522

[3] WHO. Early initiation of breastfeeding to promote exclusive breastfeeding, e-Library of Evidence for Nutrition Actions (eLENA). 2019.

[4] Hurley WL, Theil PK. Perspectives on immunoglobulins in colostrum and milk. Nutrients. 2011;3(4):442–74. doi: 10.3390/nu3040442 22254105 PMC3257684

[5] Binns C, Lee M, Low WY. The long-term public health benefits of breastfeeding. Asia Pacific Journal of Public Health. 2016;28(1):7–14. doi: 10.1177/1010539515624964 26792873

[6] Dudeja S, Sikka P, Jain K, Suri V, Kumar P. Improving first-hour breastfeeding initiation rate after cesarean deliveries: A quality improvement study. Indian pediatrics. 2018;55(9):761–4. 30345980

[7] Neovita. Timing of initiation, patterns of breastfeeding, and infant survival: prospective analysis of pooled data from three randomised trials. The Lancet Global Health. 2016;4(4):e266-e75. 10.1016/S2214-109X(16)00040-1 27013313

[8] Onalo R. Neonatal hypothermia in sub-Saharan Africa: a review. Nigerian journal of clinical practice. 2013;16(2):129–38. doi: 10.4103/1119-3077.110120 23563449

[9] Smith ER, Hurt L, Chowdhury R, Sinha B, Fawzi W, Edmond KM, et al. Delayed breastfeeding initiation and infant survival: a systematic review and meta-analysis. PloS one. 2017;12(7):e0180722. doi: 10.1371/journal.pone.0180722 28746353 PMC5528898

[10] Boerma T, Ronsmans C, Melesse DY, Barros AJ, Barros FC, Juan L, et al. Global epidemiology of use of and disparities in caesarean sections. The Lancet. 2018;392(10155):1341–8. doi: 10.1016/S0140-6736(18)31928-7 30322584

[11] Dikete M, Coppieters Y, Trigaux P, Fils JF, Englert Y, Simon P, et al. Variation of caesarean section rates in Sub-Saharan Africa: A literature review. Journal of Gynecological Research and Obstetrics. 2019;5(2):042–7.

[12] Gedefaw G, Demis A, Alemnew B, Wondmieneh A, Getie A, Waltengus F. Prevalence, indications, and outcomes of caesarean section deliveries in Ethiopia: a systematic review and meta-analysis. Patient safety in surgery. 2020;14(1):1–10. doi: 10.1186/s13037-020-00236-8 32292491 PMC7140488

[13] Organization WH. WHO statement on caesarean section rates. World Health Organization; 2015.

[14] Banapurmath C, Ramachandrappa S, Guruprasad G, Biradar SB. Is Cesarean Section a Barrier to Early Initiation of Breastfeeding. Indian Pediatr. 2013. 24382907

[15] Getnet B, Degu A, Yenealem F. Prevalence and associated factors of early initiation of breastfeeding among women delivered via Cesarean section in South Gondar zone hospitals Ethiopia, 2020. Maternal health, neonatology and perinatology. 2020;6(1):6. doi: 10.1186/s40748-020-00121-3 33298188 PMC7724884

[16] Shiferaw R, Tadesse SE, Mekonnen TC, Zerga AA. Timely Initiation of Breast Feeding and Associated Factors among Caesarian Section Delivered Mothers in Health Facilities of Dessie City Administration, North Eastern Ethiopia. Pediatric reports. 2020;13(1):1–8. doi: 10.3390/pediatric13010001 33374654 PMC7838866

[17] Mekonnen A, Shewangizaw Z. Timely initiation of breastfeeding and associated factors among mothers with vaginal and cesarean deliveries in public hospitals of Addis Ababa. 2021.

[18] MOH E. Federal Democratic Republic of Ethiopia Ministry of Health Health Sector Development Program IV October 2010 Contents. October 2010. 2014.

[19] CSA. Southern Nations, Nationalities, and Peoples’ Region. Religion [edit], 19, 10. Religion [edit]. 2007;19(28.4):10.

[20] Taha Z, Ali Hassan A, Wikkeling-Scott L, Papandreou D. Prevalence and Associated Factors of Caesarean Section and its Impact on Early Initiation of Breastfeeding in Abu Dhabi, United Arab Emirates. Nutrients. 2019;11(11). doi: 10.3390/nu11112723 31717627 PMC6893450

[21] Khanal V, Scott JA, Lee AH, Karkee R, Binns CW. Factors associated with Early Initiation of Breastfeeding in Western Nepal. International journal of environmental research and public health. 2015;12(8):9562–74. doi: 10.3390/ijerph120809562 26287223 PMC4555298

[22] Neupane M, Dhungana GP, Sharma B, Paudel D. Prelacteal Feeding and Associated Factors among Mothers of Infants Aged 6 to 12 Months in Chitwan District, Nepal: A Community Based Cross-sectional Study. medRxiv. 2021.

[23] Johar N, Mohamad N, Saddki N, Ismail TAT, Sulaiman Z. Factors associated with early breastfeeding initiation among women who underwent cesarean delivery at tertiary hospitals in Kelantan, Malaysia. Korean journal of family medicine. 2021;42(2):140. doi: 10.4082/kjfm.19.0178 32423181 PMC8010441

[24] Takahashi K, Ganchimeg T, Ota E, Vogel JP, Souza JP, Laopaiboon M, et al. Prevalence of early initiation of breastfeeding and determinants of delayed initiation of breastfeeding: secondary analysis of the WHO Global Survey. Sci Rep. 2017;7:44868. doi: 10.1038/srep44868 28322265 PMC5359598

[25] Baya E. Initiation of breastfeeding among babies delivered by ceaserian Section in Kenyatta national hospital and Pumwani maternity Hospital: University of Nairobi; 2015.

[26] Emam E, Ali AS. Factors influencing breastfeeding practice after cesarean section delivery. J Nurs Health Sci. 2017;6(5):63–70.

[27] Nisa J, Salimo H, Budihastuti UR. Factor of socio demography and obstetric that influence the timeliness of early breastfeeding in Tegal regency. J Matern Child Matern. 2017;2(2):89–99.

[28] Boakye-Yiadom AP, Nguah SB, Ameyaw E, Enimil A, Wobil PNL, Plange-Rhule G. Timing of initiation of breastfeeding and its determinants at a tertiary hospital in Ghana: a cross-sectional study. BMC Pregnancy Childbirth. 2021;21(1):468. doi: 10.1186/s12884-021-03943-x 34193067 PMC8247238

[29] Islam MA, Mamun A, Hossain MM, Bharati P, Saw A, Lestrel PE, et al. Prevalence and factors associated with early initiation of breastfeeding among Bangladeshi mothers: a nationwide cross-sectional study. PloS one. 2019;14(4):e0215733. doi: 10.1371/journal.pone.0215733 31022237 PMC6483221

[30] Habtewold TD, Sharew NT, Alemu SM. Evidence on the effect of gender of newborn, antenatal care and postnatal care on breastfeeding practices in Ethiopia: a meta-analysis andmeta-regression analysis of observational studies. BMJ open. 2019;9(5):e023956. doi: 10.1136/bmjopen-2018-023956 31152028 PMC6549640

[31] Vieira TO, Vieira GO, Giugliani ER, Mendes CM, Martins CC, Silva LR. Determinants of breastfeeding initiation within the first hour of life in a Brazilian population: cross-sectional study. BMC Public Health. 2010;10:760. doi: 10.1186/1471-2458-10-760 21143893 PMC3020684

[32] Sharma A, Thakur PS, Tiwari R, Kasar PK, Sharma R, Kabirpanthi V. Factors associated with early initiation of breastfeeding among mothers of tribal area of Madhya Pradesh, India: a community based cross sectional study. Int J Community Med Public Heal. 2016;3(1):194–9.

[33] Ahmad MO, Sughra U, Kalsoom U, Imran M, Hadi U. Effect of antenatal counselling on exclusive breastfeeding. Journal of Ayub Medical College Abbottabad. 2012;24(2):116–9. 24397070

[34] Gurung R, Sunny AK, Paudel P, Bhattarai P, Basnet O, Sharma S, et al. Predictors for timely initiation of breastfeeding after birth in the hospitals of Nepal-a prospective observational study. International Breastfeeding Journal. 2021;16(1):1–7. 34715883 10.1186/s13006-021-00431-y PMC8555201

[35] Crider C. Breast Crawl: Did You Know Your Newborn Was Capable of All This? available at https://www.healthline.com/health/breastfeeding/breast-crawl. 2020.

[36] Karim F, Khan ANS, Tasnim F, Chowdhury MAK, Billah SM, Karim T, et al. Prevalence and determinants of initiation of breastfeeding within one hour of birth: An analysis of the Bangladesh Demographic and Health Survey, 2014. PloS one. 2019;14(7):e0220224. doi: 10.1371/journal.pone.0220224 31344069 PMC6658080

[37] Kalisa R, Malande O, Nankunda J, Tumwine JK. Magnitude and factors associated with delayed initiation of breastfeeding among mothers who deliver in Mulago hospital, Uganda. African health sciences. 2015;15(4):1130–5. doi: 10.4314/ahs.v15i4.11 26958013 PMC4765405

