# Supplementary material for: Timely initiation of breastfeeding among women who gave birth by cesarean section in central Ethiopia, 2022: A cross-sectional study
Source: PLoS One. 2023 Sep 27;18(9):e0291983. doi: 10.1371/journal.pone.0291983 (PMC10529530; doi:10.1371/journal.pone.0291983)
Supplement: S1 File — (DOCX) [file pone.0291983.s002.docx]

## Questionnaire

**Name of the institution________________________________Date_______/______/_______**

**Medical record number (card number) ____________________________________________**

**Data collector name & signature _________________________ Identification code _______**

**Part I. Socio-demographic and economic characteristics related questions**

| **No** | **Questions** | **Response** | **Skip** |
| --- | --- | --- | --- |
| 101. | Mother’s age? | _____________Years. |  |
| 102. | Mother’s educational level? | 1. No formal education 2. Read and write 3. Primary school 4. Secondary school 5. College and above |  |
| 103. | Mother’s employment or occupation? | 1. Government employed 2. Non-governmental organization. 3. Private organization employed. 4. Merchant 5. House wife 6. Others specify __________ |  |
| 104. | Mother’s religion? | 1. Protestant 2. Orthodox 3. Muslim 4. Others specify__________ |  |
| 105. | Current marital status? | 1. Married 2. Single 3. Divorced 4. Widowed 5. Others Specify; _________ |  |
| 106. | Place of residence? | 1. Urban 2. Rural |  |
| 107. | Family size? | Male ____Female ____Total ________ |  |
| 108. | Educational status of your husband? | 1. No formal education 2. Read and write 3. Primary 4. Secondary 5. College/above |  |
| 109. | Household’s average monthly income? | __________________ ETB |  |

**Part II: Maternal awareness about the timely initiation of breastfeeding-related questions**

| **No** | **Questions** | **Response** | **Skip** |
| --- | --- | --- | --- |
| 201. | Have you ever heard about the timely initiation of breastfeeding? | 1. Yes. 2. No. | **If no skip to 203** |
| 202. | If yes for Q 201, from where did you get the information? | 1. Radio 2. Television 3. Health professionals 4. Neighbors 5. Others specify ______ |  |
| 203. | Did you have a breastfeeding experience? | 1. Yes. 2. No. |  |
| 204. | Timely initiation of breastfeeding is important for the newborns? | 1. Yes. 2. No. |  |
| 205. | Is colostrum (first breastmilk) helpful for your newborns? | 1. Yes. 2. No. |  |
| 206. | Did you feed the colostrum (firstbreastmilk) toyour baby? | 1. Yes. 2. No. | **If yes skip to 208** |
| 207. | If you didn’t feed the colostrum to yourbaby, what was the reason? | ____________________ |  |
| 208. | Have you given any feeding rather thanbreast milk since he/she was born? | 1. Yes. 2. No. |  |
| 209. | If yes for Q 208, what was given? | ____________________ |  |

**Part III: Maternal Obstetric Characteristics related questions**

| **No** | **Questions** | **Response** | **Skip** |
| --- | --- | --- | --- |
| 301. | Parity? | _________________ |  |
| 302. | Did you have a history of stillbirth? | 1. Yes 2. No | **If no skip to 304** |
| 303. | If yes for Q302, how many times? | _________________ |  |
| 304. | Did you have a history of early neonatal loss? | 1. Yes 2. No |  |
| 305. | Did you have a history of abortion? | 1. Yes 2. No | **If no skip to 307** |
| 306. | If yes for Q305, how many times? | _________________ |  |
| 307. | Did you get ANC service during your current pregnancy? | 1. Yes 2. No | **If no skip to 311** |
| 308. | If you get ANC service, where did you get the service? | 1. Health center 2. Hospital 3. Private clinic. |  |
| 309. | How many times did you get ANC service? | Number of ANC Visits _____ |  |
| 310. | Did you received counseling about timely initiation of breastfeeding during your ANC visits? | 1. Yes 2. No |  |
| 311. | Current pregnancy status? | 1. Intended pregnancy 2. Unintended pregnancy |  |
| 312. | Gestational age at delivery? | _________weeks | From chart review |
| 313. | Duration of labor? | _________hours | From chart review |
| 314. | What was an indication of her cesarean section delivery? | _________________ | From chart review |
| 315. | Type of cesarean section? | 1. Elective CS 2. Emergency CS |  |
| 316. | Type of anesthesia given? | 1. Spinal anesthesia 2. General anesthesia | From chart review |
| 317. | Time of delivery? | 1. Day. 2. Night. |  |
| 318. | Time taken to perform caesarian section procedure? | _______minutes/ hours | From chart review |
| 319. | Who attended the cesarean section delivery procedure? | 1. Senior obstetrician 2. Obstetrics residents 3. IESO 4. Others specify _______ | From chart review |
| 320. | Did you received post-cesarean section counseling about timely initiation of breastfeeding? | 1. Yes 2. No |  |
| 321. | Time of skin to skin contact with your neonate? | 1. Within one hour of birth. 2. After one hour of birth. |  |
| 322 | Did you get health professionals support to timely initiate breastfeeding? | 1. Yes 2. No |  |

**Part IV: Maternal Obstetric and medical complication related questions**

| 401. | Did you have any obstetric complications? | 1. Yes. 2. No. | **If no skip to 403.** |
| --- | --- | --- | --- |
| 402. | If yes for Q 401, which type of obstetric complication? (circle more than one if present) | 1. Preeclampsia 2. Eclampsia 3. APH 4. PPH 5. Sepsis 6. Others specify, _______ |  |
| 403. | Did you have any medical complications? | 1. Yes. 2. No. | **If no skip to 501.** |
| 404 | If yes for Q 403, which type of medical complication? (circle more than one if present) | 1. Chronic hypertension 2. Cardiac disease 3. Diabetes mellitus 4. HIV/AIDS 5. Others specify, _______ |  |

**Part V: Timely initiation of Breastfeeding related questions**

| 501. | **For those who gave birth under spinal anesthesia.**  When did you initiate breastfeeding for your newborn? | 1. Within one hour of birth. 2. After one hour of birth. |
| --- | --- | --- |
| 502. | How long after delivery did you start breastfeeding your newborn? | _______ minutes or _____hours |
| 503. | **For those who gave birth under general anesthesia.**  When did you initiate breastfeeding for your newborn? | 1. As soon as after recovering from anesthesia effect. 2. Not as soon as after recovering |
| 504. | How long after delivery did you start breastfeeding your newborn? | ____________________hours |
| 505. | If **option 2 selected ineitherQ 501 or 503**, what was the reason that made you delay breastfeeding initiation? | _______________________ |

**Part VI: Newborn factors related questions**

| **No** | **Questions** | **Response** | From chart review |
| --- | --- | --- | --- |
| 601. | Sex of newborn? | 1. Male 2. Female |  |
| 602. | Birth weight of newborn? | _________________________kilograms |  |
| 603. | **APGAR** score of the baby within the first minute and fifth minutes after birth? | 1^st^ minute ____________________ and 5^th^ minutes ___________________ |  |
